# Hypothalamic–pituitary–adrenal axis activity, personality traits, and BCL1 and N363S polymorphisms of the glucocorticoid receptor gene in metabolically obese normal-weight women

**DOI:** 10.1007/s12020-014-0187-0

**Published:** 2014-02-18

**Authors:** Joanna Porzezińska-Furtak, Barbara Krzyżanowska-Świniarska, Tomasz Miazgowski, Krzysztof Safranow, Ryszard Kamiński

**Affiliations:** 1 Department of Hypertension and Internal Medicine, Pomeranian Medical University, 71252 Szczecin, Poland; 2 Department of Biochemistry & Medical Chemistry, Pomeranian Medical University, Szczecin, Poland; 3 West Pomeranian Institute of Psychotherapy, Szczecin, Poland

**Keywords:** Cortisol, Copeptin, Personality traits, Glucocorticoid receptor gene, Metabolically obese normal-weight

## Abstract

We sought associations among metabolic profiles, copeptin levels, emotional control, personality traits, and hypothalamic–pituitary–adrenal axis activity in metabolically obese normal-weight young women (MONW). We assessed body composition, including fat-free mass; body fat (BF) and android and gynoid fat depots; fasting blood glucose, insulin, copeptin, cortisol (baseline and after dexamethasone), adrenocorticotropin (ACTH), triglycerides, total cholesterol, low- (LDL) and high-density (HDL) lipoproteins; and the BCL1 and N363S polymorphisms of the glucocorticoid receptor gene in 59 MONW and 71 healthy women aged 20–40 years. We also evaluated personality traits using the NEO-Five Factor Inventory and the subjective extent of emotional suppression by the Courtauld Emotional Control Scale. Compared to the controls, MONW had significantly higher insulin, cholesterol, LDL, triglycerides, and waist circumference, but lower HDL. MONW also had increased BF (>30 % of weight) and unfavorable regional fat distribution with excess android fat. The android/BF ratio was 8.29 % (MONW) versus 7.89 % (controls) (*p* = 0.005), while the gynoid/BF ratio was 31.99 versus 34.1 %, respectively (*p* = 0.008). Despite similar ACTH levels in both groups, MONW had higher cortisol levels both at the baseline (*p* < 0.001) and in the dexamethasone suppression test (*p* = 0.003). Copeptin levels and the distribution of glucocorticoid receptor polymorphisms were similar in both groups. There were also no significant differences in psychological features between MONW and controls. In conclusion, the MONW phenotype was associated with hypothalamic–pituitary–adrenal axis dysregulation, unfavorable metabolic profiles, and fat accumulation, but normal distribution of glucocorticoid receptor gene polymorphisms and copeptin levels, and no significant differences in psychological features between MONW and controls.

## Introduction

The metabolically obese, normal-weight (MONW) phenotype is associated with a high risk of insulin resistance, impaired glucose tolerance, dyslipidemia, hypertension, and excess body fat [1–3]. It has been suggested that the largest MONW group comprises middle-aged subjects in whom early intervention based on physical activity and dietary restrictions might be greatly beneficial [1, 3, 4]. However, in the general population, the MONW phenotype is commonly underdiagnosed, mainly due to youth, normal body mass index (BMI) and, typically, normal medical history [5, 6]. Moreover, early identification of MONW subjects is impeded by the lack of uniform diagnostic criteria. Earlier studies sought to simplify the complex criteria originally proposed by Ruderman et al. [7] and were based on a single measurement of insulin resistance via the hyperinsulinemic-euglycemic clamp protocol [8], fasting insulin level [9], or surrogate indices of insulin resistance such as homeostasis model assessment (HOMA-IR) [3]. In addition to normal or slightly elevated BMI, other studies proposed visceral fat >100 cm^3^ in computed tomography [2], and the coexistence of impaired glucose tolerance [10] or metabolic syndrome [11] as diagnostic criteria for MONW. However, regardless of the criteria used for diagnosis, a majority of studies found that MONW subjects frequently display a cluster of typical obesity-related cardiometabolic abnormalities [1–3, 12, 13].

Some studies performed on healthy, normal-weight subjects suggested possible associations between serum cortisol concentrations and risk for metabolic disorders [14, 15]. Hypothalamic–pituitary–adrenal axis activity is altered under stress and may be influenced by changes in individual ability to suppress certain negative emotions and personality traits [16, 17]. One of the recently identified stress markers that might also be associated with metabolic disorders is copeptin [18–20]. Copeptin is derived from the same precursor molecule, pre-provasopressin, as vasopressin (AVP) and neurophysin 2. AVP exerts a potentiating effect on corticotropin-releasing hormone (CRH), while both agents stimulate adrenocorticotropin (ACTH) secretion. There have been no previous reports evaluating hypothalamic–pituitary–adrenal axis activity and psychological parameters in MONW individuals. Therefore, we sought associations among body composition, anthropometric measurements, copeptin levels, emotional control, personality traits, and hypothalamic–pituitary–adrenal axis activity in young subjects with MONW.

## Materials and methods

### Study population

The study group comprised 130 healthy, normal-weight, Caucasian women aged 20–40 years living in the Szczecin area of Poland. Participants were randomly recruited from local electoral lists and were invited to participate in the study with a letter of invitation. The exclusion criteria were: BMI >25.0 kg/m^2^; familial hyperlipidemia; history of thyroid, cardiovascular, kidney, or liver disease; arterial hypertension; pregnancy; irregular menstruation; and any medical condition that required pharmacological treatment, including oral contraceptives taken more recently than 3 months prior to entering the study.

All the participants were interviewed to obtain past medical history. Physical examinations were performed, including measurements of blood pressure, heart rate, weight, height, and waist (WC) and hip (HC) circumference. The body adiposity index (BAI) was calculated from the following equation: BAI = HC (cm)/height (m)^1.5^—18.

Women were classified as MONW when waist circumference was >80 cm and at least two of the following criteria were met: (1) low-density cholesterol >3.0 mmol/l; (2) high-density cholesterol <1.29 mmol/l; (3) triglycerides >1.7 mmol/l; (4) glucose >5.6 mmol/l (the diagnostic criteria for metabolic syndrome); (5) total cholesterol >4.9 mmol/l; and (6) HOMA-IR >1.69 [3].

The Pomeranian Medical University Ethics Committee approved the study protocol and all the subjects gave their written informed consent.

### Assessment of body composition

Body composition, including fat-free mass, body fat (BF), and android and gynoid fat depots, was assessed by dual-energy X-ray absorptiometry (DXA) (GE Lunar Prodigy Advance; Madison, WI, USA; software enCORE version 8.1) according to the standard protocol provided by the manufacturer. Android fat depots were assessed between the lines from the top edge of the L2 vertebra to the bottom edge of the L4 vertebra. Gynoid fat depots were assessed between the lines running through the greater trochanter of the femur and the flexion gap in the knee joint.

### Biochemical analyses

Biochemical assessments included fasting blood glucose, insulin, copeptin, cortisol, ACTH, triglycerides, total cholesterol, and low- (LDL) and high-density (HDL) lipoproteins. All measurements were performed using commercially available assays. From fasting glucose and insulin levels, a HOMA-IR index was calculated using the following equation: HOMA-IR = insulin (μIU/ml) × glucose (mg/dl)/405. Participants who additionally agreed to undergo the dexamethasone suppression test were asked to take 1 mg of dexamethasone at bedtime and underwent a blood test on the following day between 7:00 and 8:00 a.m.

### Polymorphisms of the glucocorticoid receptor gene

All participants were genotyped for the BCL1 and N363S polymorphisms of the glucocorticoid receptor gene. DNA was extracted from the peripheral blood leukocytes (Master Pure™ Complete DNA Purification Kit, Illumina Inc., Madison, WI, USA) and both polymorphisms were determined by PCR–RFLP (polymerase chain reaction—restriction fragment length polymorphism) analysis according to a protocol described elsewhere [21–23].

### Psychological assessment

We applied two psychological assessment tools: the NEO-Five Factor Inventory (NEO-FFI) and the Courtauld Emotional Control Scale (CECS). The NEO-FFI is a short version of the NEO PI-R (Revised NEO Personality Inventory), a questionnaire that consists of 240 items measuring five personality traits: extraversion, agreeableness, neuroticism, openness to experience, and conscientiousness. The NEO-FFI consists of 60 items (12 items per domain), of which the participant rates statements on a five-point scale ranging from “strongly disagree” to “strongly agree.”

The CECS questionnaire comprised three subscales (anger, anxiety, and depression) with five statements for each type of emotion rated by the participant on a five-point scale. It measures the subjective extent of emotional suppression in difficult situations. The overall outcome is given as the total emotion control index [24].

### Statistical analysis

Differences between groups were evaluated by the Mann–Whitney *U* test for continuous variables and by the Chi square test with Yates correction or Fisher’s exact test for dichotomous variables. Spearman’s rank correlation coefficients were calculated to determine the relationships between continuous variables. Since the majority of variables showed a skewed distribution, they were logarithmized before performing correlation and regression analyses. Data are presented as medians and interquartile ranges.

## Results

Of 130 women, 59 were classified as MONW (on the basis of criteria used in this study) and the remaining 71 served as the control group. Baseline comparisons in anthropometric measurements, metabolic profiles, biochemical assessment, and body composition between MONW and healthy controls are shown in Table 1. Compared to the controls, MONW had significantly higher levels of insulin, glucose, total cholesterol, LDL, triglycerides, and WC, but lower HDL. MONW also had increased BF (>30 % of weight) and unfavorable regional fat distribution with excess android fat that was comparable gynoid fat. The android/BF ratio was 8.29 % (MONW) versus 7.89 % (controls) (*p* = 0.0053), while the gynoid/BF ratio was 31.99 % versus 34.1 %, respectively (*p* = 0.008).

**Table 1:** Clinical characteristics, metabolic profiles, and body composition in MONW and controls

	MONW (*n* = 59)	Controls (*n* = 71)	*p* value
	Median (IQR)	Median (IQR)	
Age (years)	33.0 (28–37)	32.0 (27–35)	0.216
Height (cm)	167.0 (162–170)	166.0 (161–169)	0.608
Weight (kg)	62.8 (56.4–66.8)	58.8 (53.4–64.8)	0.053
Body mass index (kg/m^2^)	22.27 (21.2–23.8)	21.43 (19.5–23.5)	**0.049**
Waist circumference (cm)	77.0 (71–83)	73.0 (69–78)	**0.012**
Hip circumference (cm)	97.0 (91–99.5)	94.0 (90–99)	0.232
Waist-to-hip ratio	0.8 (0.76–0.83)	0.77 (0.75–0.81)	**0.048**
Body adiposity index (%)	26.89 (25.2–29.5)	26.82 (24.2–28.3)	0.363
Systolic blood pressure (mm Hg)	118.0 (105–125)	115.0 (110–120)	0.378
Diastolic blood pressure (mm Hg)	75.00 (70–80)	78.00 (70–89)	0.464
Heart rate (beats per minute)	72.00 (68–80)	72.00 (67–80)	0.904
Body fat (% weight)	35.40 (29.1–38.4)	30.80 (25.1–34.1)	**<0.001**
Body fat (kg)	20.72 (16.7–23.6)	16.64 (13.1–21.3)	**<0.001**
Fat-free mass (kg)	37.97 (35.1–40.4)	38.84 (35.8–41.6)	0.223
Android (kg)	1.72 (1.43–2.13)	1.18 (0.95–1,64)	**<0.001**
Gynoid (kg)	6.45 (4.82–6.93)	6.27 (12.9–16.3)	0.090
Total cholesterol (mmol/l)	5.72 (4.97–6.09)	4.38 (4.07–4.58)	**<0.001**
Triglycerides (mmol/l)	0.79 (0.64–1.08)	0.70 (0.53–0.87)	**0.006**
HDL-C (mmol/l)	1.58 (1.29–1.89)	1.76 (1.48–1.99)	**0.026**
LDL-C (mmol/l)	3.34 (2.98–3.88)	2.23 (1.97–2.51)	**<0.001**
Glucose (mmol/l)	5.16 (4.72–5.38)	4.92 (4.72–5.16)	0.073
Insulin (μIU/ml)	7.50 (5.36–9.9)	4.70 (3.4–6.4)	**<0.001**
HOMA-IR	1.57 (1.17–2.25)	1.05 (0.76–1.37)	**<0.001**
Cortisol (nmol/l)	433.1 (303.5–535.2)	304.9 (232.0–413.8)	**<0.001**
Cortisol after dexamethasone (nmol/l)^a^	31.17 (27.59–35.86)	26.76 (23.45–27.59)	**0.003**
ACTH (pmol/l)	2.18 (1.80–2.79)	2.18 (2.0–3.61)	0.190
Copeptin (pmol/l)	0.84 (0.57–1.04)	0.75 (0.58–1.27)	0.676

Data are medians and interquartile ranges

Bold values are statistically significant (*p* < 0.05)

^a^ The dexamethasone suppression test was performed in 21 MONW and 18 controls

In MONW, but not in the controls, a negative correlation was found between android fat and HDL (*R* = −0.312; *p* = 0.016). Moreover, android fat correlated positively with total cholesterol (*R* = 0.280; *p* = 0.032), LDL (*R* = 0.272; *p* = 0.037), and triglycerides (*R* = 0.268; *p* = 0.04). We found no similar associations with either total or gynoid fat. Interestingly, despite similar ACTH levels in both groups, MONW had significantly higher cortisol levels both at baseline and in the dexamethasone suppression test. However, in all the subjects levels of ACTH, morning cortisol, and cortisol after dexamethasone were within the reference values. In logistic regression analysis, cortisol (OR 1.34; 95 % CI 1.08–1.67; *p* < 0.01) and %BF (OR 1.14; 95 % CI 1.05–1.23; *p* < 0.01), after adjusting for confounders, were independent predictors of the MONW phenotype.

The distribution of the glucocorticoid receptor polymorphisms BCL1 and N363S was similar in MONW and controls (Table 2). All studied genotypes followed the Hardy–Weinberg equilibrium in both cases and controls. Women with the BCL1 polymorphism (GC and GG genotypes) had lower BMI than CC carriers [21.60 kg/m^2^ (range 20.17–23.42) versus 22.71 kg/m^2^ (range 21.39–24.13); *p* = 0.023]. There were no other significant differences in the studied parameters between women with and without the BCL1 and N363S polymorphisms of the glucocorticoid receptor gene. There were also no significant differences in psychological features between MONW and controls (Table 3). The total emotion control index was positively correlated only with insulin and HOMA-IR and additionally in MONW with glucose (Table 4). However, no correlations were found between the total emotion control index and cortisol. In the entire group of studied women, there was fair to moderate agreement among three of five personality traits (neuroticism, extroversion, and openness to experience) and the total emotion control index and its components (Table 5).

**Table 2:** The distribution of glucocorticoid receptor polymorphisms BCL1 and N363S

Genotype	MONW (*n*) (%)	Controls (*n*) (%)	*p* value
N363S			
AA	54 (100)	60 (93.8)	
AG	0	4 (6.2)	0.124
GG	0	0	
*BCL1*			
CC	22 (40.7)	23 (35.9)	
GC	28 (51.9)	32 (50.0)	0.502
GG	4 (7.4)	9 (14.1)

**Table 3:** Psychological features in MONW and controls

	MONW	Controls	
CECS	Median (IQR)	Median (IQR)	*p* value
Anger	15.14 (12–18)	15.57 (13–18)	0.731
Depression	16.04 (13–18)	16.46 (14–19)	0.568
Anxiety	15.88 (12–19)	16.97 (15–20)	0.202
Total emotion control index	40.68 (34–51)	42.09 (39–54)	0.363
NEO-FFI			
Neuroticism	5.0 (3–7)	4.82 (2–7)	0.733
Extroversion	6.02 (4–8)	6.01 (5–7)	0.976
Openness	5.72 (5–7)	5.92 (5–7)	0.472
Agreeableness	5.34 (4–7)	5.57 (4–7)	0.732
Conscientiousness	6.29 (5–8)	6.32 (5–7)	0.838

CECS, Courtauld Emotional Control Scale; NEO-FFI, NEO-Five Factor Inventory; IQR, Interquartile range

**Table 4:** Correlations between total emotion control index and biochemical parameters in MONW and controls

	MONW		Controls	
	*R*	*p* value	*R*	*p* value
Glucose (mmol/l)	0.28	**0.029**	0.14	0.234
Insulin (μIU/ml)	0.71	**<0.001**	0.29	**0.012**
HOMA-IR	0.71	**<0.001**	0.32	**0.006**
Cortisol (nmol/l)	0.04	0.772	0.10	0.417
Cortisol after dexamethasone (nmol/l)	0.16	0.479	−0.30	0.220
ACTH (pmol/l)	0.03	0.875	−0.03	0.881
Copeptin (pmol/l)	−0.34	0.067	−0.22	0.361

Spearman rank correlation coefficients

Bold values are statistically significant (*p* < 0.05)

**Table 5:** Correlations between emotion control and personality traits in the entire group of studied women

	Total emotion control index		Anger control		Anxiety control		Depression control	
	*R*	*p*	*R*	*p*	*R*	*p*	*R*	*p*
Neuroticism	0.20	**0.023**	0.22	**0.020**	0.11	0.268	0.49	**<0.001**
Extroversion	−0.28	**0.002**	−0.33	**<0.001**	−0.22	**0.020**	−0.37	**<0.001**
Openness to experience	−0.25	**0.005**	−0.067	0.483	−0.25	**<0.001**	−0.20	**0.032**
Conscientiousness	0.03	0.766	−0.05	0.634	0.15	0.116	−0.16	0.490
Agreeableness	−0.01	0.898	0.18	0.060	−0.26	**0.006**	−0.07	0.101

Spearman rank correlation coefficients

Bold values are statistically significant (*p* < 0.05)

## Discussion

We found that MONW women had significantly higher BMI, %BF, android fat, and android/BF ratios but lower gynoid/BF ratios than healthy controls. Moreover, %BF was an independent predictor of the MONW phenotype. These findings strongly support the results of previous studies that MONW is closely associated with increased total adiposity as well as unfavorable fat distribution.

Studies evaluating hypothalamic–pituitary–adrenal axis activity in obesity have yielded inconsistent results. Some earlier reports demonstrated a causal relationship between hypothalamic–pituitary–adrenal axis upregulation and obesity or metabolic syndrome [14, 15, 25, 26]. In premenopausal obese women, Marin et al. [25] demonstrated that 24-h urine cortisol excretion correlated significantly with abdominal obesity. Similarly, cortisol responses to ACTH and CRH were higher in obese women with abdominal obesity than in women with peripheral fat distribution [26]. Other studies demonstrated a significant relationship between morning plasma cortisol and components of the metabolic syndrome [27]. In our study, MONW showed significantly higher morning cortisol concentration as well as higher cortisol in the dexamethasone suppression test. We also noted that morning and postdexamethasone cortisol levels positively correlated with total cholesterol and triglycerides. Moreover, cortisol levels after dexamethasone also correlated with LDL. In logistic regression, cortisol concentration was an independent risk factor for the MONW phenotype. To our knowledge, this is the first report demonstrating such associations in MONW. Our results may suggest hypothalamic–pituitary–adrenal axis hyperactivity in MONW, closely associated with unfavorable lipid profiles. Thus, it seems that cortisol might be involved in the development of some metabolic disorders, even when its plasma concentrations remain within normal range. On the other hand, recent studies have failed to demonstrate any associations between cortisol level and obesity or metabolic syndrome [28], suggesting that cortisol dysregulation may occur only in various subpopulations (which still need to be identified) of obese and/or insulin-resistant subjects. In addition, it has been demonstrated that local and systematic glucocorticoid action may be amplified by the expression of glucocorticoid receptor polymorphisms. In the general population, the BCL1 polymorphism was associated with higher glucocorticoid sensitivity [29, 30]; while the N363S polymorphism influenced the risk of coronary heart disease and was associated with higher BMI and unfavorable lipid profiles, although reports were not consistent [31–33]. There have been no earlier reports investigating the presence of glucocorticoid receptor polymorphisms in MONW. Aside from significantly lower BMI in BCL1 carriers, we did not find any associations among the presence of the BCL1 or N363S polymorphisms, cortisol level, and metabolic disorders. Moreover, the frequency of both polymorphisms investigated in our study seems to exclude their association with MONW. We found that the N363S polymorphism was present only in 6.25 % of controls, while in MONW it was not observed at all. On the other hand, the incidence of the BCL1 polymorphism was significantly higher than the incidence of MONW. Therefore, it seems neither of the studied polymorphisms are associated with the MONW phenotype.

In addition to direct relationships among cortisol, obesity, and metabolic syndrome, evidence also suggests that perceived psychological stress and personality traits may contribute to the risk of cardiovascular disease and metabolic disorders [34, 35]. In the present study, we addressed this issue in our healthy, normal-weight population by assessing basic personality traits and ability to suppress negative emotions. Overall, there were no differences in the tested psychological parameters between MONW and controls; although some correlations relating to metabolic disorders were found. The total emotion control index positively correlated with fasting glucose, insulin, and HOMA-IR. The tendency to suppress negative emotions in CECS also correlated with some personal traits: positively with neuroticism and inversely with openness and extraversion. These results may indicate that some psychological features may predispose individuals to develop metabolic abnormalities, namely insulin resistance. The multilateral connections between assessed parameters may suggest a constellation of psychological characteristics that substantially contributes to the risk for the MONW phenotype. The influence of psychological traits on stress adaptive mechanisms, hypothalamic–pituitary–adrenal axis activity, and cortisol levels may mediate this kind of reaction. In turn, this, along with the study of Abraham et al. [27], may suggest that specific trait questionnaires may be more useful than general stress questionnaires in understanding the role of stress in metabolic abnormalities typically found in MONW. However, this suggestion needs further study, as no role for psychological factors in pathogenesis of MONW has been elucidated as yet.

We could not demonstrate any significant associations among copeptin levels and metabolic parameters, anthropometric measurements, body composition, or MONW occurrence. Previous reports found positive correlations between plasma copeptin concentrations and the prevalence of type 2 diabetes, insulin resistance, and metabolic syndrome [19, 20]. However, these studies were performed on populations that significantly differed from our sample in size, ethnicity, age, weight, and adiposity. In these studies, plasma copeptin levels significantly correlated with BMI, fasting plasma glucose and insulin, HOMA-IR, triglycerides, and HDL [19]. We were unable to confirm these results, likely due to younger subjects, lower BMI, and a relatively modest sample size.

When interpreting our data, it is appropriate to consider certain limitations of our study. The main limitation is its cross-sectional design. Hence, the associations presented between independent factors and outcome variables do not necessarily represent causal relationships. We studied a relatively large sample of healthy, premenopausal Polish women with narrow BMI, BF, and age ranges. Therefore, the data presented in this study may not be applicable to general populations or other ethnicities. Second, in addition to possible inter-operator variation, there is an intra-equipment variation in DXA measurements even within equipment supplied by the same manufacturer; which may influence the reliability of measurements in population-based studies. Moreover, in some cases, an automatic scan mode does not fix the markers accurately within the studied region of interest, which requires manual corrections. To minimize such technical errors, we used the same DXA scanner and software version for the entire study duration. Additionally, all scans were analyzed by the same technician. Third, our study evaluated systemic cortisol levels and did not evaluate the role of cortisol within tissues or cortisol production rates. It has been suggested that minor changes in hypothalamic–pituitary–adrenal axis activity, inflammatory status, and psychological factors may contribute jointly to the pathogenesis of obesity and metabolic syndrome [30], and hence, also possibly to the MONW phenotype. Fourth, albeit Neo-FFI, in general, provides a quick, reliable, and accurate measure of the five domains of personality and CECS is a valuable psychometric instrument in the assessment of perceptions of psychological stressors; it is unclear whether these tools are also sensitive enough to assess which personality traits are MONW-specific, or how personality is related with the individual perceptions to metabolic stressors. Therefore, further studies are needed to elucidate these relationships. Finally, one of the diagnostic criteria used in this study was based on arbitrary assumed cut values for HOMA-IR, which represents only a surrogate method for the assessment of insulin resistance, derived from a single measurement of fasting glucose and insulin. Although the same criterion was used in earlier reports [3, 35], our study emphasizes the urgent need to develop more adequate diagnostic criteria which could be widely applied to the early identification of normal-weight subjects at risk for metabolic abnormalities.

In conclusion, we found that the MONW phenotype was associated not only with hypothalamic–pituitary–adrenal axis dysregulation, unfavorable metabolic profiles, and fat accumulation but also with normal personality traits, copeptin levels, and distribution of the BCL1 and N363S glucocorticoid receptor gene polymorphisms.

## References

[1] Ruderman NB, Brechtold P, Schneider SH (1982). Obesity associated disorders in normal weight individuals: some speculations. Int. J. Obes..

[2] Katsuki A, Sumida Y, Urakawa H (2003). Increased visceral fat and serum levels of triglyceride are associated with insulin resistance in Japanese metabolically obese, normal weight subjects with normal glucose tolerance. Diabetes Care.

[3] Conus F, Allison DB, Rabasa-Lhoret R, St-Onge M, St-Pierre DH, Tremblay-Lebeau A, Poehlman ET (2004). Metabolic and behavioral characteristic of metabolically obese but normal-weight women. J. Clin. Endocrinol. Metab..

[4] Dvorak RV, DeNino WF, Ades PA (1998). Phenotypic characteristics associated with insulin resistance in metabolically obese but normal weight young women. Diabetes.

[5] Meigs JB, Wilson PW, Fox CS, Vasan RS, Nathan DM, Sullivan LM, D’Agostino RB (2006). Body mass index, metabolic syndrome, and risk of type 2 diabetes or cardiovascular disease. J. Clin. Endocrinol. Metab..

[6] St-Onge MP, Janssen I, Heymsfield SB (2004). Metabolic syndrome in normal-weight Americans: new definition of the metabolically obese, normal-weight individual. Diabetes Care.

[7] Ruderman NB, Chisholm D, Pi-Sunyer X, Schneider S (1998). The metabolically obese, normal-weight individual revisited. Diabetes.

[8] Succurro E, Marini MA, Frontoni S, Hribal ML, Andreozzi F, Lauro R, Perticone F, Sesti G (2008). Insulin secretion in metabolically obese, but normal weight, and in metabolically healthy but obese individuals. Obesity.

[9] Molero-Conejo E, Morales LM, Fernández V, Raleigh X, Gómez ME, Semprún-Fereira M, Campos G, Ryder E (2003). Lean adolescents with increased risk for metabolic syndrome. Arch. Latinoam. Nutr..

[10] Goodpaster BH, Krishnaswami S, Resnick H, Kelley DE, Haggerty C, Harris TB, Schwartz AV, Kritchevsky S, Newman AB (2003). Association between regional adipose tissue distribution and both type 2 diabetes and impaired glucose tolerance in elderly men and women. Diabetes Care.

[11] Choi KM, Cho HJ, Choi HY, Yang SJ, Yoo HJ, Seo JA, Kim SG, Baik SH, Choi DS, Kim NH (2013). Higher mortality in metabolically obese normal weight people than in metabolically healthy obese subjects in elderly Koreans. Clin. Endocrinol. (Oxf).

[12] Shea JL, King MT, Yi Y, Gulliver W, Sun G (2012). Body fat percentage is associated with cardiometabolic dysregulation in BMI-defined normal weight subjects. Nutr. Metab. Cardiovasc. Dis..

[13] Wildman RP, Muntner P, Reynolds K, McGinn AP, Rajpathak S, Wylie-Rosett J, Sowers MR (2008). The obese without cardiometabolic risk factor clustering and the normal weight with cardiometabolic risk factor clustering: prevalence and correlates of 2 phenotypes among the US population (NHANES 1999-2004). Arch. Intern. Med..

[14] Wallerius S, Rosmond R, Ljung T, Holm G, Björntorp P (2003). Rise in morning saliva cortisol is associated with abdominal obesity in men: a preliminary report. J. Endocrinol. Invest..

[15] Phillips DI, Barker DJ, Fall CH, Seckl JR, Whorwood CB, Wood PJ, Walker BR (1998). Elevated plasma cortisol concentrations: a link between low birth weight and the insulin resistance syndrome?. J. Clin. Endocrinol. Metab..

[16] Smith SM, Vale WW (2006). The role of the hypothalamic-pituitary-adrenal axis in neuroendocrine responses to stress. Dialogues Clin. Neurosci..

[17] Savic D, Knezevic G, Damjanovic S, Spiric Z, Matic G (2012). The role of personality and traumatic events in cortisol levels-where does PTSD fit in?. Psychoneuroendocrinology.

[18] Katan M, Christ-Crain M (2010). The stress hormone copeptin: a new prognostic biomarker in acute illness. Swiss Med Wkly.

[19] Saleem U, Khaleghi M, Morgenthaler NG, Bergmann A, Struck J, Mosley TH (2009). Kullo IJ: plasma carboxy-terminal provasopressin (copeptin): a novel marker of insulin resistance and metabolic syndrome. J. Clin. Endocrinol. Metab..

[20] Enhörning S, Bankir L, Bouby N, Struck J, Hedblad B, Persson M, Morgenthaler NG, Nilsson PM, Melander O (2013). Copeptin, a marker of vasopressin, in abdominal obesity, diabetes and microalbuminuria: the prospective Malmö Diet and Cancer Study cardiovascular cohort. Int. J. Obes. (Lond).

[21] Wüst S, Van Rossum EF, Federenko IS, Koper JW, Kumsta R, Hellhammer DH (2004). Common polymorphisms in the glucocorticoid receptor gene are associated with adrenocortical responses to psychosocial stress. J. Clin. Endocrinol. Metab..

[22] Barat P, Duclos M, Gatta B, Roger P, Mormede P, Moisan MP (2005). Corticosteroid binding globulin gene polymorphism influences cortisol driven fat distribution in obese women. Obes. Res..

[23] Dobson MG, Redfern CP, Unwin N, Weaver JU (2001). The N363S polymorphism of the glucocorticoid receptor: potential contribution to central obesity in men and lack of association with other risk factors for coronary heart disease and diabetes mellitus. J. Clin. Endocrinol. Metab..

[24] Ward AM, Fall CH, Stein CE, Kumaran K, Veena SR, Wood PJ, Syddall HE, Phillips DI (2003). Cortisol and the metabolic syndrome in South Asians. Clin. Endocrinol. (Oxf).

[25] Marin P, Darin N, Amemiya T, Andersson B, Jern S, Bjorntorp P (1992). Cortisol secretion in relation to body fat distribution in obese premenopausal women. Metabolism.

[26] Pasquali R, Anconetani B, Chattat R, Biscotti M, Spinucci G, Casimirri F, Vicennati V (1996). Carcello Carcello A, Labate AM: hypothalamic-pituitary-adrenal axis activity and its relationship to the autonomic nervous system in women with visceral and subcutaneous obesity: effects of the corticotropin-releasing factor/arginine-vasopressin test and of stress. Metabolism.

[27] Abraham SB, Rubino D, Sinaii N, Ramsey S, Nieman LK (2013). Cortisol, obesity, and the metabolic syndrome: a cross-sectional study of obese subjects and review of the literature. Obesity (Silver Spring).

[28] van Rossum EF, Koper JW, van den Beld AW, Uitterlinden AG, Arp P, Ester W, Janssen JA, Brinkmann AO, de Jong FH, Grobbee DE, Pols HA, Lamberts SW (2003). Identification of the BclI polymorphism in the glucocorticoid receptor gene: association with sensitivity to glucocorticoids in vivo and body mass index. Clin. Endocrinol. (Oxf).

[29] Stevens A, Ray DW, Zeggini E, John S, Richards HL, Griffiths CE, Donn R (2004). Glucocorticoid sensitivity is determined by a specific glucocorticoid receptor haplotype. J. Clin. Endocrinol. Metab..

[30] Lin RC, Wang XL, Morris BJ (2003). Association of coronary artery disease with glucocorticoid receptor N363S variant. Hypertension.

[31] Di Blasio AM, van Rossum EF, Maestrini S, Berselli ME, Tagliaferri M, Podestà F, Koper JW, Liuzzi A, Lamberts SW (2003). The relation between two polymorphisms in the glucocorticoid receptor gene and body mass index, blood pressure and cholesterol in obese patients. Clin. Endocrinol. (Oxf).

[32] Ahola K, Pulkki-Råback L, Kouvonen A, Rossi H, Aromaa A, Lönnqvist J (2012). Burnout and behavior-related health risk factors: results from the population-based Finnish Health 2000 Study. J. Occup. Environ. Med..

[33] Melcescu E, Griswold M, Xiang L, Belk S, Montgomery D, Bray M, Del Ben KS, Uwaifo GI, Marshall GD, Koch CA (2012). Prevalence and cardiometabolic associations of the glucocorticoid receptor gene polymorphisms N363S and Bcl1 in obese and non-obese black and white Mississippians. Hormones (Athens).

[34] Branth S, Ronquist G, Stridsberg M, Hambraeus L, Kindgren E, Olsson R, Carlander D, Arnetz B (2007). Development of abdominal fat and incipient metabolic syndrome in young healthy men exposed to long-term stress. Nutr. Metab. Cardiovasc. Dis..

[35] Bednarek Tupikowska G, Stachowska B, Miazgowski T, Krzyżanowska-Świniarska B, Katra B (2012). Evaluation of the prevalence of metabolic obesity and normal weight among the Polish population. Endokrynol. Pol..

